# Post-implementation perspectives on smokefree prison policy: a qualitative study with staff and people in custody

**DOI:** 10.1093/eurpub/ckab075

**Published:** 2021-08-27

**Authors:** Ashley Brown, Danielle Mitchell, Kate Hunt

**Affiliations:** Institute for Social Marketing and Health, University of Stirling, Stirling, UK

## Abstract

**Background:**

A comprehensive smokefree prison policy (SFPP) was introduced in Scottish prisons from November 2018, reflecting concern about inequalities in occupational exposures to second-hand smoke (SHS) and tobacco-related harms among people in custody (PiC). We aimed to address a gap, whereby few studies have sought to understand SFPP from the perspectives of people living and working in prisons.

**Methods:**

As part of a comprehensive evaluation, focus groups and interviews with staff (n = 99) and interviews with PiC (n = 23) were conducted 6–9 months post-implementation of SFPP in Scotland. Data were analysed using the framework approach.

**Results:**

Our study found that new restrictions on smoking had been widely accepted by PiC, after a period of adjustment which was less troublesome than participants had anticipated. Benefits of the SFPP for the safety and comfort of staff and PiC who were no longer exposed to SHS, and additionally for the health of PiC who were now smoking-abstinent, were widely acknowledged. Drawbacks of the SFPP, such as difficulties managing without tobacco and use of alternatives (e.g. e-cigarettes and changes in use of illegal drugs), were also reported. Contraband tobacco was not reported to be a major problem following prisons becoming smokefree.

**Conclusions:**

The findings strengthen evidence that SFPPs can be implemented without causing major disruption and highlight the need for removal of tobacco to be underpinned by careful planning, partnership working and ensuring the availability of support for smokers. Experiences from Scotland may be of interest and some comfort, internationally for jurisdictions considering smoke-free rules in prisons.

## Introduction

The WHO Framework Convention on Tobacco Control^1^ requires countries to protect people from exposure to second-hand smoke (SHS) in work and indoor public spaces. When smokefree policies, were introduced in Scotland from March 2006, prisons had partial exemption in the legislation: people in custody (PiC) were permitted to smoke in designated rooms (their cell) and outdoors. In July 2017, the Scottish Prison Service (SPS) and Scottish Government announced their intention to strengthen smokefree policies in prisons,^2^ in light of new evidence on SHS levels in Scotland’s prisons.^3^ The new comprehensive (indoor and outdoor) smokefree prison policy (SFPP) was implemented in November 2018.

Very few studies (see evidence from USA,^4^^,^^5^ Australia^6^ and Taiwan^7^) have qualitatively explored the perspectives of people working or living in prisons with recently implemented SFPP, and none has comprehensively investigated these across a prison system.

Findings from these existing studies vary, potentially reflecting differing penal contexts and implementation strategies. Nonetheless, the published literature highlights possible challenges of prohibiting smoking in prison (e.g. contraband tobacco^4^^,^^5^^,^^7^ and misuse of NRT^6^) and factors which may facilitate (e.g. effective communication strategies, good smoking cessation/abstinence support^5^^,^^6^) or impede, a more successful transition to SFPP. This study seeks to build on and enhance the very limited number of previous qualitative studies, by exploring experience of the recently implemented SFPP from the perspective of staff and PiC, providing evidence on opinions on SFPP, success factors and positive/negative consequences once the policy had embedded. The data were collected 6–8 months post-implementation of SFPP, as part of the most in-depth evaluation of SFPP to date [the Tobacco In Prisons study (TIPs)]. Previous papers from TIPs^8–10^ and a complementary study of e-cigarette use in prison^11^ report views of staff and PiC, prior to implementation of the SFPP using different participant samples.

## Methods

Ethical approval was granted by the SPS Research Access and Ethics Committee and University of Glasgow, College of Social Science Research Ethics Committee (ref: 400150214).

### Research settings, sampling and recruitment

SFPP was introduced in Scotland’s prisons from November 30, 2018. It prohibits PiC from smoking tobacco in any areas of prisons; staff have been prohibited from smoking or using e-cigarettes within prison grounds from 2008. The transition to SFPP was underpinned by a wide-reaching implementation strategy, including enhancement of existing smoking cessation support (see^12^) and new rules allowing PiC to purchase and use e-cigarettes in designated areas (see^11^).

For this study, we analyze data from staff focus groups in Scotland’s 14 ‘closed’ prisons, involving 95 participants in total. A focus group conducted in Scotland’s open prison was excluded from this analysis, since the issues staff raised were very distinct, as PiC spend some time in the wider community and have access to tobacco whilst outside the prison. Points of contacts in prisons were asked to arrange one focus group, ideally comprising up to eight staff in various work roles and a mix of staff who did or did not smoke and/or vape. In addition, four staff with leading roles in the implementation of SFPP at local (prison) level were interviewed, to capture their perspectives. The combined staff focus group and interview sample (99 participants) comprised: 75 men and 24 women, 83 never- or ex-smokers and 13 current smokers (3 did not report their smoking status) and 26 who reported ever using e-cigarettes. Most participants were SPS staff (a few were healthcare staff); most (*n* = 66) were prison officer grade and had worked in prisons for 11+ years (*n* = 62).

Interviews with PiC were conducted in six (closed) prisons, selected to represent a range of prisons in terms of size and population (men/women, young people/adults, individuals who were untried/convicted and serving shorter/longer sentences). We chose in-depth interviews, rather than focus groups, with PiC for methodological, ethical and practical reasons (e.g. allowing PiC to speak freely on a potentially sensitive topic, without concern about the complex social and interpersonal dynamics among PiC). Through the staff points of contact, researchers asked that PiC with varied characteristics that might have a bearing on experiences of SFPP (i.e. sex, remand/convicted status and sentence length) were invited to participate. Of the 23 PiC interviewed (18 men, 5 women; 12 aged ≤40, 11 aged 41+), all were convicted, 13 serving sentences of 4 years or less and 10 of 4+ years. All were former smokers; although this was not an explicit inclusion criterion it reflects the previously high rates of smoking in prisons prior to smokefree policy.

### Data collection

Data collection took place 6–9 months post-SFPP. Focus groups with staff ranged in size (3–14 people). Two joint interviews were conducted at the request of the participants; the remaining interviews were one-to-one. Interviews/focus groups were conducted with only A.B. and/or K.H. present, in a room/area in the prison where participants could not be easily overheard.

Topic guides for interviews and focus groups largely covered similar topics for PiC and staff, informed by the study objectives, existing literature, research team discussions and input from staff within the prison service. They included: participant background; opinions of SFPP; perspectives on living/working in a smokefree prison (including successes/challenges and positive/negative consequences); compliance and enforcement of SFPP and lessons learned.

### Data analysis and reporting

De-identified transcripts were thematically analysed using the framework approach, following a process described elsewhere.^11^ A.B. led on developing a thematic framework based on close reading of transcripts, study objectives and existing literature. To synthesize and distil material prior to interpretation and abstraction, data were organized under themes and summarized (by A.B. and D.M.) and displayed in a grid format (row=focus group/interview and column=theme) in NVivo 12. A.B. reviewed all summaries to check consistency and interpretations. A.B. led the detailed thematic analysis by comprehensively and systematically searching framework grids and reviewing data excerpts, to identify and compare perspectives and experiences of SFPP. Using an iterative process, different dimensions of the data were organized into themes and sub-themes, which were then structured to produce a coherent narrative. All authors agreed final themes based on reviewing transcripts/substantial involvement in data collection. Extracts, selected to evidence and illustrate key findings, are attributed to participants (staff/PiC) using a serial number (letter randomly allocated to each prison for this paper and participant number), an indication of smoking/vaping status (S = smoker, ExS = ex-smoker, NS = never-smoker, V = vaper, ExV = ex-vaper and NV = never-vaper) and, for staff, whether they worked in a prison officer (PO), managerial (MGR) or other role. Supporting extracts are presented in tables 1–4.

**Table 1:** Acceptance and support for the smokefree policy

i. Acceptance of the smokefree policy		
Q1	Staff	D3 (PO-ExS-ExV): And I think everyone, they [PiC] all seemed to just accept it, and get on with it. Which we all were grateful for.
Q2	PiC	**I: What’s your thoughts on the ban now it’s been about six months or so since it’s been in place…?** B3 (M-ST-ExV): Part of life. …. it is what it is, it’s there, it’s not going away, get on with it now.
ii. Positive/negative opinions of smokefree policy		
Q3	Staff	N6 (PO-S-S-V): [Everybody’s] working environment deserves to be smoke-free whether it is a jail or an office or whatever.
Q4	PiC	G2 (F-LT-ExV): I think people should be allowed to smoke, it’s like your human right…Even if it was an outdoor thing…when you go out for exercise you can smoke or something…
Q5	PiC	C4 (F-ST-V): …you’ve got a hundred lassies [women]…all with their own mental health problems. You’re taking away the one thing that…that could be the difference.
Q6	PiC	L2 (M-ST-V): I really thought it [smokefree rules] was a great thing, not just for myself, health-wise, but, also, it’s not fair on officers when they come into a smoky situation.

**Table 2:** Implementation success factors and challenges

i. Ease of transition to the smokefree policy		
Q7	PiC	B1 (M-ST-ExV) …. people were anticipating a lot of the things they thought were going to happen… But [smokefree policy] came in then everything that people thought was going to happen never happened…it just went…quite peacefully. There was no major uproar…
Q8	Staff	K1 (MGR-ExS-V): I think that we were expecting a lot of issues, a lot of problems, and there's not been, there's genuinely, it's been really smooth.
ii. Management of transition to the smokefree policy		
Q9	Staff	H13 (MGR-ExS-NV): …it was a well-planned move. It was. No doubt about it. And it was done really well.
Q10	PiC	L4 (M-LT-V): If you're talking about instituting the whole thing from scratch, I think they done it all right. Yes, I think they [SPS] done it all right. My judgment.
iii. Communication and engagement		
Q11	PiC	M1 (F-LT-V): Every month there was a new poster. Four months to go. Three months to go. And we were like, “We know, we know!”.
Q12	Staff	P2 (MGR-NS-NV)…for me the communications were the key thing and that was local communications, national communications. There was a variety of different models….[publicising the smokefree policy on] the canteen sheets, so making people aware…[it was] coming. The posters, the focus groups. [A staff member from] HQ came…and did various groups with prisoners and, kind of, started explaining with the vapes and I think communications, you just can’t get enough communication. But it came to a stage where a few prisoners were saying…”you’re not banging on about this smoking thing again!” That’s when you realise you’ve, kind of, reached a stage where they know what’s happening.
Q13	Staff	M6 (PO-NS-NV): I would have like to have seen the vapes come in a wee bit faster….
		M5 (PO-NS-NV): It’s true there wasn't enough, there wasn't enough time [between the introduction of rechargeable e-cigarettes and the date of the ban]…I think that was just because of the…procurement side of things… It…would have been much, much, better for us to get the vapes in an awful lot earlier.
iv. Collaborative working		
Q14	Staff	A5 (PO-NS-NV): …Huge amounts of work done by the addictions teams, the gymnasium, the work party officers. Everybody’s contributing…we’re asking their [PiC] opinions. And I think that had a massive input. A8 (UNKNOWN): I think you're probably right…getting their [PiC] opinions, and involvement, and ideas, as well. A4 (PO-S-EXV):Yeah, it goes a long way, doesn’t it.
Q15	Staff	R1 (MGR-NS-NV) …it’s been a massive change and I’m really proud of it. …The team working was the best I’ve ever done with the NHS…. real collaboration, real laughs, real shared concerns, real action plans. “Oh I’ll pick that up”, “oh actually I’m someone that can do that”, rather than… Not that it’s always that things tend to be forced, but you’ve got your remits, they’ve got their remits. Even though you’ve got the same goals, it doesn’t mean that you’re necessarily going to work in a way that synergises everyone. But this did.
v. Smoking abstinence/cessation support		
Q16	Staff	E4 (MGR-EXS-NV): I think the NHS…the smoking cessation team that was in here did a great job… So I think they contributed a big part. Massive.
vi. Prior implementation of smokefree policies in public places		
Q17	Staff	B1 (PO-S-V): I think also the smoking bans in pubs and things like that, people are probably are more aware of the fact that smoking is changing. Like over the last maybe five years, people have known that you can’t smoke in public places and things like that. And there’s more of a change happening. So, I think it’s easier to accept [smokefree prison policy].. when something else is changing regarding smoking…I would say that maybe helped them basically.

**Table 3:** Tobacco-related benefits/drawbacks

i. Elimination of SHS: health and comfort		
Q18	Staff	C1 (MGR-ExS-NV):I think it’s [smokefree policy] fantastic. **I:In what way?** C1 (MGR-ExS-NV):Well, one, I don’t have to smell the smoke. Two, I don’t have to breathe in smoke. Three, the air is certainly a bit fresher, a bit cleaner. And generally, overall, it’s to the benefit of everybody’s health that we’re not having to breath in smoke or smell it. **I: What do others think about that?** C4 (PO RES-NS-NV):Yes, I agree. A great improvement to our working life because the halls [residential areas] are quite enclosed, so, you know, you [would] smell it off your clothing when you went home.
Q19	PiC	C5 (F-ST-V): …the room’s so much cleaner because before you would have to, sort of, clean the walls and things like that when you went in. Like, you’d spray cleaning stuff on the walls and it would just run, like, yellow, see with the nicotine. And even, like, you’d go into a room, you wouldn’t actually notice it but…until you clean it and you’re like, oh…that’s horrible…before it would…like, you go out the room, you come back in you’re like…it…stinks of stale smoke. Whereas, like, it’s a lot fresher. It’s a lot nicer.
ii. Smoking abstinence/cessation: perceived health benefits		
Q20	PiC	M2 (M-ST-V): Well to start off with I thought it [smokefree policy] was a joke. But obviously after a couple of weeks you adjust to a life without cigarettes…you start to feel a bit better…with your breathing. Your lung capacity, your fitness, everything starts picking up.
Q21	PiC	**I: what about in terms of how you feel in yourself health wise, are you seeing any differences?** M3 (M-ST-ExV): Massive difference, massive. I can play football again, I can go to the gym and I’m not out of breath. I’ve noticed a difference in my skin complexion and my eyes. Aye, I just feel a lot better, aye. If you’d seen me when I came in you would know what I was talking about. I feel a lot better.
Q22	Staff	I5 (Other role-Ex-V,): …. although it [stopping smoking] wasn’t their choice, they’re actually feeling the benefits, better breathing health-wise, more energy, things like that. So overall so far the feedback we’ve got has actually been really positive. Even from the guys that are just like, “oh I’m still going to smoke when I go out”, but see at this moment in time, I feel great…. So there’s a lot that are really positive about it [smokefree rules].
iii. Smoking abstinence/cessation: unintended adverse consequences		
Q23	PiC	C3 (M-ST-V): I did enjoy smoking when I was in prison, it's one of the small things that I had…was having a smoke, and a coffee… just to kind of, de-stress, kind of…relax. And now it's been taken away, I feel like my anxiety has increased a lot.
Q24	PiC	O1(M-LT-V): Because tobacco is not around…it's causing a lot of aggro, as well…a lot of more fights now, than what there used to be, and more arguments…. more tension.
Q25	PiC	M4 (M-LT-V): I feel better. I’ve got angina and stuff, so I do, I feel better. The thing is, and this is the catch…because I have so much more money, I buy myself more treats [from the prison shop]. So, I’ve put on [weight] since [stopping smoking].
iv. Facilitators/barriers to continued smoking abstinence/cessation post release		
Q26	PiC	L3 (M-ST-V): What’s the use of going back to it [smoking] if you’ve been off it that long. What’s the use of picking it up when you get out after two year? That’s a total waste.
Q27	PiC	C2 (M-ST-V): I miss it, yes. …. It doesn’t get any easier…you’re thinking, you’re like, the day you get out [of prison] to get a cigarette…
Q28	PIC	G1: it’s easier not having smoking in jail, because I wouldn’t go to vaping, but outside, if you feel like that, you can just go to the shop, and you’re not going to just buy a packet of fags for about a tenner, have one fag and chuck it away, so it does worry me that I’m going to go back to it…

**Table 4:** Use of alternative substances

i. E-cigarettes		
Q29	PIC	B4 (M-ST-V) …: if they didn’t get put [e-cigarettes] in, there would’ve been riots, 100 per cent. That made it a little bit better.
Q30	PiC	B2 (M-LT-V): [E-cigarettes are] better for you anyway. At least there’s something there…Rather than just cold turkey, man.
Q31	PiC	**I: …do you think it was the right thing for them to start selling vapes in prisons?** M1 (F-LT-V): That’s quite a hard…It’s like yes and no. Because we don’t know the long term effects, but I think it’s stopped maybe people causing riots and things like that. I think it’s cut down on a lot of things, a lot of negative things that could have happened if nothing was put in place…
Q32	Staff	G4 (PO-NS-NV):I think the transition’s been better than we anticipated…the vaping…seems to have made it a lot easier. … I think it would have been really difficult for someone who smoked 40 years and… **I1:There’s a few others nodding there.** G5 (PO-NS-NV):Yeah. I’d agree with that. I think there was a lot of anxiety before it came in…And I think the vaping, the smoking cessation sessions certainly helped.
Q33	Staff	J5 (PO-ExS-NV): I’ve got to admit, it’s gone better than I imagined it’d ever go, with the vapes coming in. Is it going to be the right decision? We’ll probably not know that for 30 years, will we? … Until we see in maybe 30 years what the outcome of the vapes are, we’re not going to really know if it’s been a good thing or a bad thing, because I feel it’s still too early.
Q34	Staff	O1 [PO-NS-NV] …there's still obviously concerns, regarding the vapes…It seems like an appeasement thing, from the SPS, in order to bring the smoking ban in easier for them. Also, I don't know how much health effects against staff that the e-cigarettes can have on us, as well. And obviously, they're [PiC] adapting them, as well, for the NPS [‘new’ psychoactive substances] … too. So, basically in my opinion, we shouldn’t have any vapes.
Q35	PiC	M5 (M-LT-V): For me personally, they're taking us off the smoking, so we can't get the smoke, but they're giving you something else. And to me, that doesn’t make sense. If they want us to be smoke free, then make it smoke free. It's like, being [addicted to] heroin and saying, no, give us that, but here’s methadone. It's the same sort of thing. It defeats the purpose, taking them off one thing and giving them…to me, that’s madness.
Contraband tobacco and misuse of NRT		
Q36	Staff	Q1 (MGR-ExS-ExV) I’m sure there’s probably some tobacco in here, but it’s such a rare find for us. So either they’ve hid it so well that we don’t see it, we don’t smell it, or it isn’t here. I would suggest that [tobacco’s] not here because people have taken to the vapes.
Q37	PiC	C2 (M-ST-V)…. there’s obviously spells when you get tobacco in the halls, you know what I mean, obviously everything comes in, drugs, tobacco, you know what I mean, but not very often [for tobacco].
Q38	PiC	M2 (M-ST-V):…what’s the point in bringing something in to the prison, especially if it’s [tobacco] strong smelling. It’s going to go out throughout the prison as people’s noses are going to start picking up like dogs, like [makes sniffing noise]. And the prison guards are going to smell it as well. So it’s quite a noticeable smell.
Q39	PiC	M4 (M-LT-V): The people who want to smuggle things in…don’t want to smuggle in big lumpy bits of tobacco. They want to smuggle in drugs or wee tiny things or stuff like that. **I: Easier to get in or worth more?** M4 (M-LT-V): It’s worth a lot more and far easier to get in.
Q40	PiC	C3 (M-ST-V): When the ban first came in…People were getting [NRT] patches, and smoking the patches. I don't know why, but apparently that’s what people were doing. **I:Is that still going on, do you think?** C3 (M-ST-V): I don't see people with patches anymore…I don't think patches get used at all now, it's just vapes, that’s the only sort of replacement that I hear about now, is vapes.
ii. Psychoactive substances/illegal drugs		
Q41	Staff	L3 (PO-S-NV): Are they transferring their nicotine habit and replacing it by using other substances as a coping strategy? We don’t know but I have seen, over the last six months to a year, more and more people [under the influence of NPS]… […] L1 (PO-ExS-NV): …. NPS would probably have come in anyway, but the methods of using it [using e-cigarettes], we’ve given them an easier… L3 (PO-S-NV):Opportunity….. […] L1 (PO-ExS-NV)…. Whereas beforehand, people were using their lighters [for drug taking]
Q42	PiC	L1 [M-LT-ExV]: They cut them [e-cigarettes] open and use the element out of them to smoke ‘legal highs’ [NPS], but you’re going to get that anywhere you go. Any jail you go to. **I: And [was] NPS…an issue before the smoking ban?** L1 (M-LT-ExV): Aye. Well, you must have seen the news and all that. It’s in every jail … it’s…horrible, man. Horrible, horrible thing, man. I don’t understand how anybody can smoke that shit. Aye, you’re going to always get somebody abusing something in the jail. If they can’t get a lighter, they always find some mad way of getting a light.
Q43	PiC	M5 (M-LT-V) …I think…there's quite a big thing about illegal highs [NPS], and things. And there's a few problems in the hall because of that…So if they cut out, even just cutting out that [e-cigarettes], in that hall alone, it would be totally different. Because then, when they're not being able to do that [because lighters are difficult to obtain], so they're not getting all uptight and angry. If it's not there, it's not there, know what I mean. There's guys getting into debt over it, there's guys getting hurt over it. There's officers getting hurt over it.
Q44	Staff	D2 (MGR-ExS-NV): [Because of NPS]. You’ve got people [having hallucinations]…swimming up a section [residential area]. A guy lying on the floor, like the floor’s electric.. And this all sounds make-believe, they’ve no idea what they're taking. [.…] they are that close to dying. And then, two days later, they're back on it [NPS].
Q45	PiC	G3 (M-LT-ExV): See to be honest, it [smokefree policy] has kind of helped me a lot because it has kind of taken all my thing like drugs and everything away because I only ever smoke weed and I smoke it with tobacco, so no tobacco I don’t smoke weed.

## Results

We present findings on two key areas: factors contributing to successful implementation and benefits and challenges of SFPP.

### Perspectives on policy implementation and success factors

Both staff and PiC indicated that, after a relatively brief adjustment period, the SFPP was widely accepted by PiC and had become the norm in prisons (table 1; Q1 and Q2). However, as before the ban,^8^^,^^9^ SFPP remained more popular among staff than PiC post implementation. Popularity amongst staff was partly explained by the immediate impact of SFPP in reducing SHS levels in prisons (table 1; Q3). Concerns about restricting PiC’s smoking choices and about the necessity and consequences of SFPP continued to be voiced in interviews with PiC (table 1; Q4 and Q5) and, to a lesser degree, by some staff. However, some PiC were, on balance, supportive of the SFPP because of perceived benefits for them personally and for non-smokers who were now protected from SHS (table 1; Q6).

The introduction of the SFPP was generally perceived to have been less troublesome than staff and PiC had anticipated (table 2; Q7 and Q8), and prior fears about the possibility of significant disorder (e.g. ‘riots’) in prisons had not materialized. Several factors which might have aided the relatively smooth implementation of SFPP were identified. First, the transition to SFPP was reportedly well managed by the SPS at local and national levels (table 2; Q9 and Q10). The decision to stop tobacco sales several weeks prior to the implementation date, and permitting local policies for the removal of tobacco, were highlighted as important aspects of the implementation strategy, since they increased the likelihood of PiC cutting down smoking in anticipation of SFPP.

Second, good communication and engagement with PiC and staff were perceived as important to the successful implementation of SFPP. ‘Countdown’ posters around prisons were generally said to have ensured widespread awareness of the impending SFPP (table 2; Q11), although it was acknowledged that some PiC had not taken notice of information. Hence, considerable efforts were also made to engage with PiC to understand their views, identify potential solutions to problems, signpost to cessation support and get feedback on issues such as e-cigarettes (table 2; Q12). However, some staff said they would have appreciated earlier communication about the detailed implementation strategy (e.g. introduction of rechargeable e-cigarettes) (table 2; Q13).

Third, collaboration with and input from a range of stakeholders was perceived by prison staff to have been instrumental to success, given the scale and complexity of the task of prohibiting smoking among PiC. This included partnership working across health and justice services (NHS and SPS), engagement with staff and PiC on SFPP and broad acceptance of SFPP among staff and PiC (table 2; Q14 and Q15).

Fourthly, ready availability of smoking abstinence/cessation support in prisons was considered essential for SFPP implementation preparation (table 2; Q16). There was discussion in some staff groups about how this support might need to evolve under smokefree rules. For example, one group discussed integrating support for nicotine addiction with other health promotion activities, to take a more holistic approach to improving PiC’s health going forward.

Finally, the de-normalization of smoking in many contexts following the 2006 legislation prohibiting smoking in most public places in Scotland was perceived by prison staff to have aided implementation of SFPP (table 2; Q17).

### Perceptions of benefits and challenges of SFPP

The perceived benefits and challenges of SFPP were discussed in relation to three main themes, as discussed below.

#### Elimination of SHS

The widespread elimination of SHS from prisons was viewed as a significant gain for the health of staff (and also PiC) (table 3; Q18). In some instances, staff discussed experiencing fewer symptoms, such as asthma, a sore throat and eye irritation, following SFPP. Staff, and some PiC, typically commented on improved sensory experiences, such as no longer smelling tobacco smoke in the air, improved appearance and cleanliness of the prison and their clothes no longer smelling of stale smoke (table 3; Q18 and Q19). Long-serving staff reflected on how markedly SHS in prisons had reduced over several decades following successive tightening of smoking restrictions. Some expressed disappointment that progress to entirely smokefree prisons had not been quicker.

#### Smoking abstinence/cessation

PiC generally acknowledged general health benefits of stopping smoking, and some reported improvements in their own health (e.g. in fitness, breathing) following implementation of SFPP (table 3; Q20, Q21 and Q22). However, negative physiological and psychological impacts of SFPP were also raised by PiC and staff, particularly for new arrivals with insufficient funds to purchase (rechargeable) e-cigarettes to help them adapt to a smokefree environment. While these negative impacts reportedly reduced over time for some PiC, others continued to struggle with the SFPP. Some still sought effective strategies for managing nicotine dependence and coping with common problems such as poor mental health or low mood (table 3; Q23) or filling time in prison. A few PiC said that mood changes attributed to smoking abstinence contributed to tension and conflict in prison (table 3; Q24) and a few reported unwanted weight gain (table 3; Q25).

The data also highlighted potential opportunities and challenges for extending the benefits of SFPP when abstinent smokers are released from prison. Some suggested the experience of health or financial benefits from not smoking in prison may increase people’s motivations to give up smoking long-term, and strengthen beliefs that there is something to lose from smoking relapse (particularly for individuals on longer sentences) (table 3; Q26). There were some suggestions that use of NRT or e-cigarettes may help some individuals to avoid smoking relapse after leaving prison. Conversely, the association of smoking with pleasure, comfort and relaxation, a history of co-use of tobacco and cannabis and returning to environments where tobacco is available/smoked were cited as important potential barriers to remaining smokefree post-release (table 3; Q27 and Q28).

#### Use of alternatives to tobacco

Both participant groups spoke about the use of alternatives to tobacco, as discussed below.

##### E-cigarettes, NRT and illicit tobacco

It was reported that most former smokers had switched to vaping following the SFPP. Levels of support for e-cigarettes in prisons remained stronger in PiC than staff, who voiced more diverse views about the advantages and disadvantages of e-cigarettes in prisons. In both samples, benefits of e-cigarettes for implementation of SFPP (table 4; Q29, Q30 Q31 and Q32) and supporting abstinent smokers in the prison population were discussed.

However, staff reported that e-cigarettes had also brought challenges relating to: uncertainties about any health risks of exposures to e-cigarette vapour; misuse of e-cigarettes for drug taking and creation of extra problems for staff to manage, such as when PiC run out of e-liquids (table 4; Q33 and Q34). Other potential risks relating to e-cigarettes in prisons (raised by both staff and PiC) included concerns about continued nicotine addiction amongst PiC (table 4; Q35), user safety and cost. Some staff voiced doubts about whether e-cigarettes would be of net benefit in the long-term and worried that hard-won gains to health from SFPP may be undermined by widespread e-cigarette use.

Data from both staff and PiC suggested that use of illicit tobacco was not a significant problem within Scotland’s (closed) prisons post-implementation (table 4; Q36 and Q37) (although there was reported to have been a period immediately post-implementation when stockpiled tobacco was in circulation). The data suggested that the scarcity of illicit tobacco may reflect: general acceptance of SFPP among PiC; availability of e-cigarettes; risks that illicit smoking will be detected and a potentially lower risk-return ratio for smuggling contraband tobacco compared with other items (e.g. illegal drugs) (table 4; Q38 and Q39). The data also suggest that misuse of NRT products (patches) in smokefree prisons is not a major concern (table 4; Q40).

##### Psychoactive substances/illegal drugs

SFPP was perceived by some staff and PiC to have contributed to changes in the use of ‘new’ psychoactive substances (NPS), which had already been identified as a problem within the prisons several years prior to the legislative change.^13^ These included changes to the method of ingesting NPS (using repurposed e-cigarettes) and suggestions that some PiC may have taken NPS for pleasure/escapism in the absence of tobacco; as a cheaper alternative to vaping and/or as a replacement for previous consumption of smuggled cannabis (which had become increasingly difficult since the sale of tobacco, rolling papers and lighters stopped in prison) (table 4; Q41–Q43).

Both PiC and prison staff spoke about the adverse impacts of use of these unpredictable substances by PiC (table 4; Q44). Concerns about NPS use among PiC included risks for users of acute injury or death and impairment of cognitive functioning, with some participants describing people behaving like ‘zombies’ after continued use of NPS. For bystanders, particularly staff, concerns centred on risks from passive exposure to NPS or from assault or accidental injury by those under the influence.

By contrast, in some instances SFPP was said to have contributed to a decrease in other illegal drug use in prison, by reducing ease of access to materials such as lighters and rolling papers (table 4; Q45).

## Discussion

Our findings, collected as part of a comprehensive evaluation of smokefree prisons,^3^^,^^8^^,^^10^^,^^11^^,^^14^ suggest SFPP has been widely accepted by PiC and prison staff in Scotland, and the removal of tobacco had been less troublesome organizationally than either group expected. Benefits of the SFPP for the health, safety and comfort of staff following the wholescale reduction of SHS, verified in post-implementation measurements,^14^ and the health of PiC no longer smoking were acknowledged. However, participants also reported difficulties of enforced smoking abstinence for certain groups (e.g. new admissions) and use of alternatives to tobacco in prisons (e.g. e-cigarettes, change in use of illegal drugs). The findings support our earlier studies^8^^,^^9^ that showed that support for SFPP is higher overall among prison staff than PiC and that opinions and experiences of SFPP are complex; it is possible for individuals to be positive about some dimensions of smokefree rules, but negative about others, as similarly illustrated in our contemporaneous surveys of prison staff and PiC.^15^

The findings contribute new knowledge about SFPP in several ways. First, they enhance understandings of SFPP, for example by highlighting ways in which some PiC may (re)frame smoking abstinence in prison as beneficial for themselves and others, while also confirming the need to be attendant to the physiological and psychological challenges of enforced smoking abstinence. Second, in contrast to other studies,^6^^,^^16^ contraband tobacco and misuse of NRT were not reported to have been major problems in prisons in Scotland at the time data were collected. These findings may be explained by high levels of vaping among PiC in Scotland as an alternative to tobacco, in contrast to other jurisdictions which have reported problems with illicit tobacco markets^5^^,^^7^^,^^16^^,^^17^ or NRT misuse.^6^ Third, although there were a few suggestions from PiC that smokefree rules were contributing to tensions or conflict in prisons, a striking finding from the study is that the introduction of SFPP had been less troublesome organizationally than many PiC and staff expected, reportedly causing no significant disorder in any prisons. This corroborates reports of SFPPs in Australia and the USA,^6^^,^^17^ and contradicts common media portrayals.^18^ Finally, our study strengthens understanding of successful factors for creating smokefree prisons by being the first to comprehensively investigate the perspectives of prison staff and PiC across a prison system post-implementation. The findings provide deeper insight into the role of factors such as extensive planning work; comprehensive communication and engagement with stakeholders and supporting PiC to abstain/quit smoking in successfully implementing SFPP (see also^6^^,^^19^). Scotland’s experience also highlights the importance of collaborative working between health and justice services, and, we believe, the benefits of access to findings from ongoing, independent research to inform strategies (e.g.^3^); all findings from TIPs were shared at the earliest opportunity with the multi-sector team responsible for smokefree implementation. The findings will be of interest to jurisdictions considering SFPP. Lessons from implementing SFPPs in Scotland can also support further public health achievements for PiC.

In relation to e-cigarettes in smokefree prisons, our novel findings are mixed and discussed in more detail elsewhere.^11^^,^^20^ Perceived benefits for SFPPs were widely discussed by staff and PiC. However, many also voiced concerns about potential risks of e-cigarettes for users, staff (bystanders), and the prison system. These will be key issues for jurisdictions which allow the sale of e-cigarettes to weigh up when planning for SFPP. Strategies for minimizing risks from e-cigarettes are likely to be beneficial if the decision to sell e-cigarettes in prisons is taken in other countries, including learning from Scotland’s novel guidance about ways to support PiC who wish to cut down or stop vaping.^11^^,^^21^

These data were collected in the final phase of the most in-depth evaluation to date of the implementation of a SFPP, conducted across an entire prison estate at three points in time: pre-announcement of plans to implement SFPP, in the lead up to implementation and post-implementation. In this paper, we have included the perspectives of staff in every (closed) prison in Scotland and perspectives of PiC from six prisons which collectively house diverse populations. The size and composition of the samples enabled a range of views and experiences to be captured, and so expand understandings of smokefree prisons considerably. Another strength is that data were collected using well-established and robust methods, 6–9 months post-implementation when staff and PiC had been able to adjust to the SFPP and possibly had a clearer sense of its benefits and drawbacks.

In relation to limitations, some perspectives (e.g. of people on remand) may be missing from this study, either because data were not collected from PiC in every prison in Scotland or due to self-selection bias. Future studies could investigate the views of PiC who are never-smokers. This part of the TIPs study was not designed to quantify potential impacts of the smokefree policy; ongoing work is analysing prison and health service data on outcomes of interest in an economic analysis of the SFPP.

The findings highlight the need for ongoing investment to maximize the long-term gains of SFPP. In future work we intend to explore how best to support PiC to remain smokefree post-release.

## Conclusion

The findings substantially strengthen international evidence that SFPP can be implemented, and maintained, without major organizational disruption. Factors promoting success include: careful system-wide planning, engagement and communication strategies, collaboration across relevant organizations and services, and supporting PiC to abstain or quit smoking, including through evidence-based interventions.
